# Peto’s paradox revisited: black box vs mechanistic approaches to understanding the roles of mutations and promoting factors in cancer

**DOI:** 10.1007/s10654-022-00933-x

**Published:** 2022-12-13

**Authors:** Allan Balmain

**Affiliations:** Helen Diller Family Comprehensive Cancer Center, University of California, San FranciscoSan Francisco, CA 1450 3rd Street94143 USA

**Keywords:** Mutations and cancer risk, Cancer epidemiology, Tumour Promotion, Cancer prevention

It is not often the case that scientific papers describing mathematical models of cancer, especially those including complex equations, can be described as “entertaining”, but this is certainly the case for several seminal papers written by Richard Peto. The purpose of this short review is to revisit one such paper, written in 1984, entitled “The need for ignorance in cancer research” [1].

In this particular article, which thankfully did not include the equations, Peto first discussed implications arising from statistical analyses of the incidence of cancer in humans and mice, identifying questions that came to be known as “Peto’s paradox”. Armitage and Doll had in 1954 published a detailed study of human cancer incidence, concluding that the rate increased in proportion to the 6th power of age [2]. In 1975 Peto carried out a mouse study that involved repeated treatment of mice with a mutagen (benzpyrene) for many months, also showing that the cancer incidence rate increased as a power of the duration of exposure to the carcinogen [3]. These data were widely interpreted as supporting a model of cancer, both in mice and humans, as being caused by a sequential series of around 4–6 mutations due to repeated long term exposure to agents that damage DNA. The paradox arose from the observation that cancer incidence and mortality in mice and humans appear to be independent of body size and longevity, in spite of huge differences in the number of cells in each species, and the length of time during which target cells may be susceptible to transformation [4]. Humans have about 2–3000 times more cells than mice, and also live about 30 times longer, providing myriad more opportunities to accumulate the mutations that are thought to underlie cancer development in both species. Human cells and tissues must therefore have developed mechanisms to deal with exceptionally high numbers of mutations, or have evolved protective strategies to deal with potentially dangerous cells that could become cancerous at an early age and thus prevent reproduction.

A second theme discussed in the 1984 review was a more philosophical analysis of the relative merits of epidemiological and experimental mechanistic approaches to identification of the major causes of human cancer. He referred to epidemiology as the ‘black box’ approach, as the predictions made by this method do not require any knowledge of the genetic or biological mechanisms by which cancers arise (hence the “ignorance” in the title of the article). He concluded that in spite of this ignorance, the bulk of the information available on cancer causation had come from black box rather than mechanism-based research. The present review will focus largely on Peto’s paradox and the cancer models that came from the black box approach vs experimental cancer models in mice–both are worth revisiting in the light of new published data that have helped to shed light on these long-standing questions.

## Has Peto’s paradox now been resolved?

Almost 50 years have elapsed since Peto carried out the work that started the debate on the basis of species-specific differences in cancer incidence and mortality [3], and almost 40 years since the appearance of the article that is the subject of this review [1]. Several recent publications have made significant contributions to our understanding of the questions raised by Peto decades ago. First, Vincze et al. [5] demonstrated by analysis of over 100,000 zoo animals from 191 different species that the underlying premise of the work carried out on mice and humans applies more generally to this broad range of species: cancer incidence is indeed independent of body size and adult life expectancy. Moreover, the studies on zoo animals provided important information on cancer causation: cancer mortality was clearly associated with diet, as carnivores had the highest cancer-related mortality among all species examined. These data have important implications because of the epidemiological association of meat consumption and a high fat diet on cancer incidence in human populations [6].

Other publications more directly addressed the underlying assumption made by Peto that humans, as large animals with a long life span, should accumulate more mutations in normal somatic cells than are seen in similar cells from mice. The technologies and conceptual advances that made it possible to answer this question included new approaches to genome sequencing on an unprecedented scale, and using very small tissue biopsies. Such studies were pioneered by Inigo Martincorena, Peter Campbell, and colleagues at the Sanger Institute, who first demonstrated that human eyelid skin from multiple individuals exhibited a very high number of mutations due to sunlight exposure [7].

Deep sequencing of normal tissue biopsies provided an ideal opportunity to determine whether mutation numbers really do increase with age across a wide range of mammalian species with different lifespans. Accordingly, Martincorena et al. carried out whole genome sequencing of 208 intestinal crypts from 56 individual animals from 16 mammalian species, in an attempt to uncover differences in mutation burdens or mutational processes across species with widely varying patterns of body size, longevity and cancer risk [8]. Crypts are clonally derived from single stem cells and accumulate mutations with age, providing the ideal scenario for cross-species comparisons.

The results appeared to be definitive: the somatic mutation rate per genome per year in normal mouse intestine was 796, but the equivalent figure for humans was 47. However because humans live about 30 times longer than mice, the end of lifespan mutational difference was only about threefold between these two species. Interestingly, variation in mutation rate was inversely correlated with lifespan, but not significantly with body mass, suggesting that animals with very large body mass, and correspondingly high numbers of cells at risk of transformation, must have evolved biological mechanisms that keep mutations in check and prevent the emergence of tumours. Others have speculated on the nature of these mechanisms, which include more efficient DNA repair systems in large animals, expansion of the number of genes encoding tumour suppressors such as the p53 gene, of which 20 copies exist in African elephants [9], or other possible mechanisms (for review see [10]). The mutations were largely attributable to endogenous mutational processes, rather than to chance exposure to exogenous mutagens, suggesting that the mutational “clocks” in different species are geared to accommodate variation in lifespan, presumably to limit the impact of high mutational load on species survival and reproduction.

These two papers by Vincze et al. and Cagan et al. provide support for the underlying basis of Peto’s paradox, but we still have a long way to go to uncover the mechanisms that allow human (and mouse) tissues carrying such high mutational burdens to survive cancer-free for so long. The mouse mutational burden data in the Cagan et al. paper [8] were from intestinal tissue from untreated animals allowed to live out their their normal lifespans. We presently have no information on the spontaneous rate of intestinal tumour development in these animals that would enable us to examine the relationship between individual mutation burden and cancer risk. The study carried out by Peto [3] involved 950 mice (all female) that underwent skin treatment with a potent mutagen for many weeks. No controls were included in this study to estimate background spontaneous tumour incidence, although historical data suggest that this is normally very low. It is therefore very difficult to compare normal tissue mutation rate, or total mutational burden, with cancer risk in the absence of more comprehensive data sets. Such data sets have been generated over the last few years by sequencing large numbers of mouse and human normal and tumour samples, and are providing new possibilities for getting to the roots of Peto’s paradox.

## Is the mutation-centric view of cancer correct?

The data in the Cagan et al. paper [8] allow us to conclude that mice accumulate mutations at a higher rate than humans, and therefore individual mouse cells may acquire the requisite number of cancer-causing mutations in a much shorter time period. At face value, this interpretation would support the largely mutation-centric view of cancer as in the classical “Vogelgram”, whereby each stage of cancer development requires a mutation in a particular pathway, eventually culminating in the emergence of the winner cells that become fully transformed [11]. The beauty of this model is that it fits nicely with the Armitage-Doll view of human cancer development as a series of around 6 rate-limiting steps [2], a conclusion widely interpreted to mean that cancer goes through a relatively small number of distinct stages before emergence of complete malignancy. The nature of these stages was never defined, but it was tempting to conclude that each stage may be caused by a specific genetic alteration, eventually leading to the acquisition of a sufficient number of genomic changes, in the correct combinations, and in the correct order, to allow tumour formation.

**Fig. 1:**
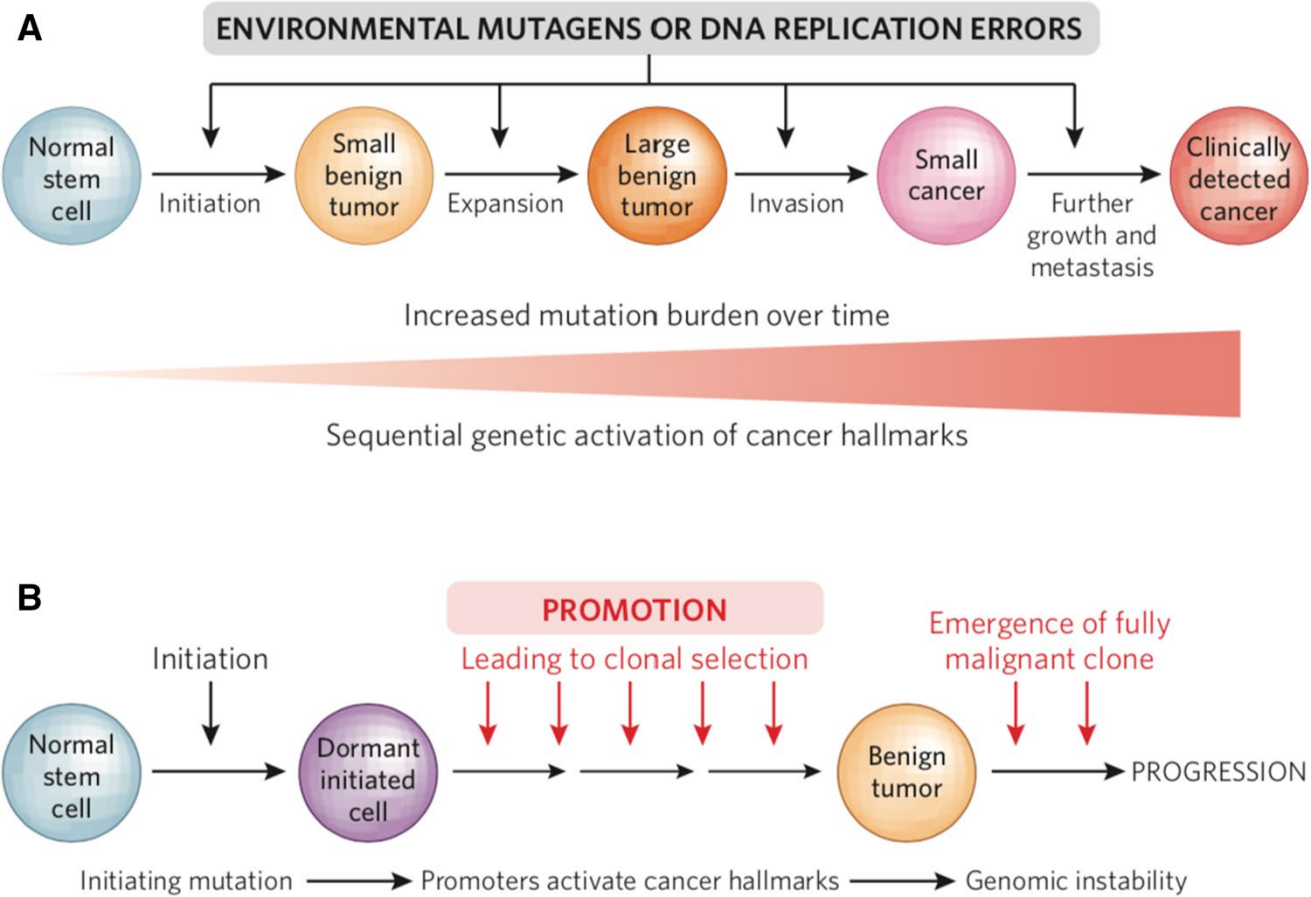
**A** The sequential mutation model for cancer development compatible with the original age-incidence data from Armitage and Doll [2] suggesting around 6 “stages”, each associated with a mutational event, and acquisition of cancer hallmarks such as self sufficiency in growth factors, resistance to cell death, recruitment of new blood vessels (angiogenesis), and ability to invade and metastasize. **B** The two stage clonal selection model, originally developed using mouse models [16–18], by which initiation is a accomplished by a single mutation, generating dormant initiated cells that are dependent on exposure to a tumour promoting agent or wounding in order to clonally expand. Tissue regeneration caused by TPA or wounding induces many of the cancer hallmark properties including increased growth factor production, angiogenesis, invasive behaviour, and immuno-evasion. Chronic treatment with a promoter is necessary to ensure clonal expansion, and initiated cell clones lose their competitive advantage if promotion is stopped. Progression involves acquisition of independence from continued promoter treatment, through genomic alterations that lead to genetic instability and loss of tumour suppressor gene activity

This model (Fig. 1A) was welcomed with open arms by the genetics/genomics community because of the advances in genome sequencing and cancer biology that led to identification of many genes, numbering in the hundreds, that are recurrently mutated in human cancers [12]. Many of these genes have been called “cancer drivers”, first because some can drive the functional attributes of cancer cells when introduced into completely normal mouse or human cells [13] and secondly because they show evidence of clonal selection in a wide range of cancer types [12, 14]. A comforting symmetry was reached with the publication of the “Hallmarks of Cancer” review by Hanahan and Weinberg in 2000, who identified 6 major hallmark pathways that are disrupted during development of human cancers [15]. These included self sufficiency in growth control, resistance to anti-growth signals, ability to evade apoptosis, as well as to generate an efficient blood supply through angiogenesis, and finally, propensity to invade and metastasize to distant sites in the body, where they can replicate indefinitely. These advances in genetics and biology seemed to dovetail perfectly with the purely statistical analysis of deaths due to cancer leading to the multistage/sequential mutation model based on the Armitage and Doll data [2].

A problem with this conclusion is that studies of cancer in mice had suggested decades ago that the rate-limiting step in cancer development is NOT the simple accumulation of mutations in cancer driver genes. Using a mouse skin model of multistage carcinogenesis, Berenblum, Mottram, and others showed that cancer development occurs in two operationally defined stages accomplished by a single low dose treatment with an “initiator” (coal tar) followed 1–2 weeks later by repeated treatment with an irritant promoting factor (croton oil) [16, 17]. The promotion had to come *after* the initiation event, otherwise tumours did not develop. It was subsequently shown that the initiation event was essentially permanent, as an insertion of an interval of between 20 weeks to over 1 year between initiation and the start of promotion demonstrated that cells capable of giving rise to tumours were still there after one year, and remained responsive to the promoting factor [18]. The conclusion was that initiation generates latent or dormant tumour cells, but that these *“ …latent tumour cells are only demonstrable by subsequent promoting action, which converts them into morphological tumours*.” (Fig. 1B).

These conclusions regarding the permanence of the initiated state have generally stood the test of time, and were replicated in the 1970s and 1980s using specific carcinogens as initiators, such as dimethylbenzanthracene (DMBA), or promoters (e.g. the active factor in croton oil: 12-O-tetradecanoyl-phorbol-13-acetate, or TPA) [19, 20]. In this laboratory, we have also repeated the original Berenblum/Shubik experiments (Rose Yun Li et al., submitted), with essentially the same results as reported in 1949. We carried out whole genome sequencing of skin carcinomas induced by the standard protocol in which promotion takes place starting 2 weeks after initiation, and the “delay” protocol, with promotion of cells that had been initiated 6–12 months previously. The results showed that the “aged” initiated cells had the same cancer driver mutations, DMBA mutational signatures, and range of total mutational burdens, as those that were promoted shortly after initiation. Some carcinomas from the “delay” protocol had 40–50,000 mutations carrying the DMBA signature. In other words, cells carrying the thousands of mutations genome-wide that were induced by the single carcinogen treatment, including highly potent oncogenic *Ras* mutations, survived intact for a significant proportion of the mouse lifespan without being removed by apoptosis or immune clearance, but also without generating any tumours or other obvious pathological changes in the tissue. These data agree with previous studies in the 1980s that led to the identification of the initiating event in this model as a carcinogen-specific mutation in the *Hras* proto-oncogene [21–23], and the demonstration that an activated version of this single gene, when introduced directly into the skin using a viral vector [24] or using a transgenic approach [25], recapitulated what had been predicted by Berenblum and Shubik: the cells carrying the mutated oncogene remained latent in the skin over several months, and only gave rise to visible tumours after repeated exposure to the tumour promoter TPA, or to a wound stimulus. The conclusion that promotion is the rate-limiting step in carcinogenesis when mice are exposed to promoters subsequent to activation of a single strong oncogene, has therefore been supported by several different experimental approaches, over several decades, in different laboratories.

## How many mutations are really required for cancer development?

At face value, these mouse data lead to the conclusion that one mutation is enough, when followed by wounding or exposure to a promoter, for development of premalignant tumours, although clearly additional genomic events are required, even in mouse models, for progression to full malignancy. However this seems to be at odds with the huge amounts of sequence data demonstrating the presence of hundreds of recurrent, putative driver mutations in human as well as chemically induced mouse tumours, including those that are premalignant([12, 14, 26], Rose Yun Li et al., submitted). So how many mutations are really needed for mouse or human cancer development? The initiation and clonal selection model is in apparent disagreement with the human epidemiology discussed above, suggesting that incidence increases in proportion to the 6th power of age and therefore involves multiple stages, each requiring its own causal genetic or biochemical change. However the human epidemiology data can also be interpreted using 2-stage models, some incorporating stochastic effects or clonal selection [27–30]. These models are more intuitively understandable in terms of the results of experiments on mice demonstrating the concept of initiation and promotion proposed in the 1940s [16–18]. While it is conceptually appealing to consider that multiple genetic events may be required for sequential activation of cancer hallmark properties (Fig. 1A), this is in fact not necessary if we consider that tumour promoter treatment mimics a chronic wound healing state, during which all of these hallmarks are naturally activated [31] (Fig. 1B). Nevertheless, in spite of this convergence of theoretical and experimental approaches in favour of a two stage model, the field has doggedly pursued the notion that sequential accumulation of multiple mutations, induced either spontaneously or by environmental carcinogens, is the main cause of human cancer.

Our thoughts on how many real cancer driver mutations are necessary for tumour development may require re-evaluation in the light of the evidence showing that normal cells and tissues have many cancer-associated mutations, but show no cancer-related pathology. Some recently published studies on other mouse models of carcinogen-induced, genetically initiated, or spontaneously arising mouse tumours have begun to shed light on these questions, and in addition have stimulated renewed interest in promotion as a crucially important step in development of human cancers.

## Different roles for mutagenic and non-mutagenic carcinogens in cancer risk

The US National Toxicology Program has investigated the possible carcinogenic effects of chemicals in the environment to which humans are exposed, in an attempt to identify true carcinogens and their mechanisms of action. They tested a large number (over 600) of known or suspected human carcinogens for carcinogenic effects by chronic exposure of rodents (mice and rats) to each agent individually using the 2 year rodent bioassay. Whole genome sequencing was carried out of a large number of mouse tumours of the lung, liver, kidney and stomach, induced by about 20 of these chemicals in various risk categories, with the goal of identifying mutational signatures [32] that could be used to establish their roles in human cancer etiology. A detailed comparison of the genomes of tumours induced by these chemicals identified two broad categories [33]. Three of the chosen chemicals clearly acted as mutagens, and produced tumours with a high mutation burden, as well as mutational signatures indicative of exogenous chemical exposures. The remaining 17 chemicals, however, induced tumours with very low mutation burdens, without any evidence of novel mutation signatures beyond what is characteristic of endogenous mutational processes. The genomes of these tumours were in fact very similar to spontaneously arising tumours in the same tissues in the same mouse strain. These data therefore suggest that the majority of known or suspected human carcinogens do not appear to act as mutagens. Their role in carcinogenesis may therefore be simply to accelerate the appearance of tumours by activating pre-existing, but latent, spontaneously initiated cells. In other words, it would appear that these environmental chemicals acted as promoters rather than as initiators.

An additional study from the Sanger Institute addressed the question why human populations in certain countries or regions of the world are at high risk of cancer development, while the risk in other countries was extremely low [34]. They sequenced esophageal cancers from individuals living in 8 different countries where there is a tenfold spread in the risk of developing this tumour type. No “smoking gun” mutational signature was found, that could have pointed to a specific carcinogen in high risk regions, as the sequences of all esophageal carcinomas tested were essentially indistinguishable. This is exactly what would be expected if the main environmental risk factor was a non-mutagenic promoter rather than a strongly mutagenic carcinogen. Parallels with the above discussion of the distinct roles of mutagens and promoting factors in the mouse skin model are obvious, and were highlighted in a recent review [35].

## A new view of normal tissue clonal architecture in relation to cancer risk

The finding that many normal human tissues have a high proportion of cells carrying somatic mutations, including cancer drivers, raises fundamental questions about the selection processes that lead to outgrowth of early neoplastic lesions under the influence environmental or dietary promoting factors. We now have to take account of a universe of mutations that arise during ageing, or in response to environmental exposures or lifestyle factors, that create evolutionary bottlenecks resulting in clonal selection. The original findings by Martincorena et al. on skin [7] were followed by a flood of fascinating publications on a wide range of human tissues including lung, colon, esophagus, kidney, endometrium and and blood (for review, see [36], revealing that mutations and clonal selection are common features of ageing in human tissues. Many of the mutations enriched in normal tissues are also seen in cancers, but others appear to be selected only in normal tissues under specific evolutionary constraints. For example, liver samples from human patients with fatty liver disease frequently have mutations in FOXO1, CIDEB and GPAM, three master regulators of lipid processing and storage, but similar mutations are not significantly enriched in hepatocellular carcinomas from human patients [37]. Importantly, similar effects on clonal architecture are seen in both mouse and human tissues, for example in the skin and esophagus, where cancer associated mutations in the p53 tumour suppressor gene, as well as in *Notch*, *Fat*, and other genes associated with cell adhesion and cell–cell competition, are commonly selected [38]. However, as noted for mutations in the *p53* gene by Doug Brash and colleagues many years ago [39], these cancer-like mutations fail to cause obvious changes in overall tissue histology, which appears completely normal. Many of the clonally selected mutations found in cancers, and therefore thought to be necessary for selection within tumours, may have pre-dated the occurrence of a rate limiting cancer driver mutation, and in effect act as passengers, or at best facilitators, rather than drivers. It is possible that a small number of critical mutations play the main causal role in disruption of tissue architecture, leading to the morphological and growth changes associated with early neoplasia.

## Integration of black box and mechanistic approaches to cancer prevention

In his 1984 review, Peto extolled the virtues of the “black box” strategy for analysis of cancer causation, which involves searching for simple correlations between human exposures or lifestyle factors and the incidence of different types of cancers. Nobody can doubt that these seminal epidemiological studies established the irrefutable links between the environment and cancer, identifying critically important causes such as smoking, obesity and certain types of viruses. Peto’s assertion was that this approach was successful in spite of the complete ignorance of the mechanisms by which these factors act, while the mechanistic strategy, which favors developing a deep understanding of how an agent works before coming up with preventive measures, would take too long, leading to too many deaths before we work it all out.

The emphasis on these extremes is of course very artificial, and clearly what is required is a combination of both approaches if we are to develop a realistic strategy for cancer prevention at multiple levels–primary, secondary and tertiary. A good example of this is how we have dealt with the smoking epidemic and its relationship to cancer. The first strong epidemiological links between smoking and cancer were published in 1950 [40, 41], although several statistical associations had been published starting as early as 1928 (reviewed in [42]). These initial black box links were followed by the demonstration that smoke contains thousands of chemicals, at least 60 of which are known carcinogens and mutagens [43]. Other studies however showed that extracts of cigarette smoke [44, 45], or of industrial air pollutants [46] also contained chemicals that could act as promoting agents in mouse skin. Of these two mechanistic advances, i.e. the demonstration that cigarette smoke has both mutagenic and promoting activities, only the former was followed up systematically, culminating in identification of precise mutational signatures showing exactly how tobacco carcinogens cause mutations in DNA [32]. The emphasis on mutational loads naturally fed into the multistage/sequential mutation model of lung cancer development, as this conformed exactly to what would be expected if the tumours only required a certain number of mutations to reach the fully transformed state. In contrast, the role of promotion has been at best an afterthought, in spite of the experimental evidence suggesting that non-mutagenic processes may play an important role in lung cancer development [44–47].

The compartmentalization of these different disciplines–epidemiological observations and mechanistic approaches, has arguably delayed the development of a more complete understanding of how cancers originate. For example, several observations made by the epidemiologists were difficult to explain in terms of the sequential mutation model. The dose–response relationship between risk of cancer and the number of cigarettes smoked per day is linear [48], as is the relationship between the number of cigarette “pack years” smoked and the total number of signature mutations in lung cancers [32]. However smoking **cessation** rapidly decreases cancer risk, and this effect becomes more pronounced over time, leading to a paradox articulated by Yoshida et al. [49]: “*Of two people who smoked the same lifetime number of cigarettes, why the one with longer duration of cessation should have lower risk of lung cancer is difficult to explain if tobacco induces carcinogenesis exclusively *via* increased mutation burden.”*

While observations on cancer risk after smoking cessation are indeed difficult to reconcile with the mutation-centric model of cancer, they appear to be readily interpretable in terms of initiation-promotion or 2 stage models. Promoting factors in cigarette smoke likely stimulate outgrowth of clones adapted to conditions of inflammatory stress, by virtue of the presence of certain driver mutations. The removal of the source of this stress will immediately begin to affect clonal evolution within the normal lung, leading to the reduction or disappearance of potentially malignant clones, and emergence of alternative clones that repopulate the lung epithelium in a more favorable tissue microenvironment. Many studies of the mouse skin model have shown that reducing the dose or frequency of exposure to tumour promoters has a very strong effect on tumour incidence, and cessation of treatment can lead to the regression of a proportion of already established early stage lesions. Integration of epidemiological and mechanistic approaches may help us to avoid future increases in cancer incidence due to ignorance of the mechanisms by which new factors in the environment may impact promotion, rather than just acting as mutagens. Ironically, smokers are now being urged to switch to vaping, as this is perceived to be non-mutagenic and therefore should not cause cancer. However several studies have already shown that vaping is associated with respiratory disease and inflammation, and may therefore function as a tumour promoter [50, 51]. It is possible that in the coming decades we may experience a new wave of lung cancers due to vaping, if indeed promotion is the rate limiting step for development of this disease. Recent data on links between air pollution and lung cancer in non-smokers support this possibility, as a combination of epidemiological, molecular, and mechanistic studies highlight the role of pollutants as promoters of lung cancers worldwide [52]. It would be regrettable if ignorance of these possible mechanisms results in dependence on future black box epidemiologists to identify a strong link between vaping and lung cancer.

## Analysis of promotion mechanisms for cancer prevention

Promoters may play a more important role in cancer etiology than was previously appreciated, but the mechanisms by which they act are unclear and likely influence different pathways in different tissues. These processes will be investigated as part of the CRUK/NCI PROMINENT initiative to address the question of *“how cells and tissues maintain “normal” phenotypes whilst harbouring oncogenic mutations and how they transition to become a tumour”* (https://cancergrandchallenges.org/teams/prominent)*.*

This Grand Challenge question is essentially a reformulation of Peto’s original paradox, asking how human tissues deal with the inevitable accumulation of mutations during ageing. The possible involvement of promoting factors as a rate-limiting cause of cancer, with strong implications for prevention, were also alluded to by Peto in his 1984 review [1]. He stated that:

”There are other lines of evidence that suggest that alteration of a normal cell into a cancer cell requires not only the ‘early’ changes that can be brought about by the action of a DNA-damaging chemical………but also other ‘late’ changes…….which can be brought about by classical ‘promoters’.” He further states that “…half an order of magnitude reduction in the rates at which these ‘late’ processes occur……would knock 70% off the cancer problem…”.

Similar sentiments were voiced by John Cairns in 1982, in a summary of the discussions at a conference on mechanisms of tumour promotion [53]:

*“If we want to prevent people from having cancer, we should be directing our attention more to the promoters in our environment than to the initiators, because it is only by removing the promoters that we can hope to benefit everyone.”*

The hope raised by these initiatives is that emphasis on inhibiting or reversing the development of cancer based on a deeper mechanistic understanding of the promotion stage will bear fruit and, as predicted by Peto, Cairns and others decades ago, have a lasting impact on cancer prevention.
